# The impact of consumer positive personality on the purchase behavior of smart products

**DOI:** 10.3389/fpsyg.2022.943023

**Published:** 2022-09-14

**Authors:** Dan Li, Dengke Yu

**Affiliations:** School of Public Policy and Administration, Nanchang University, Nanchang, China

**Keywords:** positive personality, purchase behavior, consumer knowledge, purchase intention, perceived income, smart product, structural equation model

## Abstract

While the consumption of smart products is continuously increasing, it is essential to explore the trigger mechanism of consumer behavior in respect of smart product purchase. In this scenario, we aim to investigate the impact of consumers’ positive personality on the purchase behavior. We constructed a structural equation model based on the partial least square method and tested our hypotheses on the basis of data analysis. The data were collected by conducting a survey of 326 Chinese consumers. We found two affecting paths from consumers’ positive personality to smart product purchase. First, consumer knowledge promoted by positive personality raises purchase intention and, in turn, stimulates purchase behavior. Second, consumers’ positive personality improves perceived income, which determines actual purchase behavior. This study deepens our understanding of the trigger mechanism of smart product purchase behavior and enriches the consumer behavior theory.

## Introduction

Currently, in China, the consumption of smart products is increasing rapidly. As the Zero Power Intelligence Group reported in April 2022, China has become the world’s largest smart home market consumer, accounting for 50–60% of global market share. According to its prediction, China will ship 240 million smart home devices in 2022, worth 651.56 billion yuan (Zero Power Intelligence Group, 2021). Smart phones and tablets are sought after among Chinese young people. Various enjoyable, fashionable, and smart products are getting more of their attention. More wearable smart devices, smart education, and home products are being developed and arrive in the market, contributing to China’s social transition and sustainable growth (Lv et al., 2019). Inspired by the flourishing practice, Chinese scholars have started to pay more attention to the trigger mechanism of smart product purchase behavior.

In terms of purchase behavior, previous studies on the supply side highlight the roles of corporate image and product characteristics. For example, Zhao et al. (2022) explored the effect of cosmetic advertising on brand loyalty and consumer purchase behavior. Similarly, Amoako (2022) measured the effect of corporate brand equity on purchase behavior, and Yin et al. (2022) demonstrated that origin reputation and brand reputation significantly affect consumers’ willingness to and behavior of purchase. Moreover, Kulkarni and James (2022) investigated the effects of corporate identity, product brand image, product design, and origin on the purchase of electronic products. Zhao et al. (2021) verified the positive relationship between product pricing, packaging, and consumer purchase behavior.

Similarly, scholars have put much effort to construct the link between consumers’ behavior of purchasing smart products and the characteristics of technology and products. For instance, Henkens et al. (2021) measured the effect of smartness on consumer wellbeing that reflects as self-efficacy and technology anxiety. Woo and Kim (2022) examined the effects of brand and message framing on consumers’ evaluations and purchase intentions of smart healthcare clothing. Wang and Chen (2018) argued that most consumers lack sufficient knowledge about the technology of smart products, and so they often generate the purchase intention on the basis of the information about functional characteristics or selling prices. Some other scholars also emphasized the influence of social and environmental factors. For example, Mou and Shin (2018) found that smart product social popularity would directly affect consumers’ trust, perceived quality, and perceived value and indirectly affect the decision of product purchase. The study of Hsieh and Lee (2021) also showed that media richness (social cues) and parasocial interactions (social role) are key determinants affecting the establishment of trust, perceived usefulness, and perceived ease of use, which, in turn, affect the attitude and purchase intentions of smart products. Schill et al. (2019) provided empirical evidence of their finding that environmental concerns positively affect consumers’ intentions to purchase smart home objects. Only a few scholars have concerned consumers’ emotional purchase behaviors connected with individuals’ subjective characteristics. For example, Sun et al. (2021) examined the impact of various personality factors, such as attention to social comparison information, need for uniqueness, and quality consciousness on the purchase intention for iPhone.

According to the aforementioned literature, there is an obvious limitation in the theory of consumer psychology and behavior related to the purchase of smart products. In order to fill the gap, we have conducted a survey to measure the impact of consumers’ positive personality on the intention and behavior of smart product consumption. In this study, we aim to explore the affecting paths from two aspects: First, we assume that consumers’ positive personality would stimulate the intrinsic demand for smart products and generate the intention to purchase, and second, we argue that consumers’ actual purchase behavior not only depends on the intention but also is determined by consumers’ perception of income, which restrains or expands the budget of consumption. Perceived income is regarded as a variance that positively associates with individuals’ positive personality. In our research design, the structural equation model is used to test the hypotheses.

The marginal contribution of this study to the literature is twofold. First, we built a novel integrated framework to link the relation between consumers’ individual core intangible capitals (including positive personality and knowledge) and purchasing psychology (including intention and behavior). The framework deepens our understanding of the trigger mechanism of smart product purchase and provides the possibility to develop a new theory for the cooperative governance of individual intangible capitals. Second, it would arouse and attract more scholars to develop new theories for customer relationship management in the field of smart product marketing.

The remainder of the article is structured as follows: A literature review on individuals’ positive personality and purchase behavior of smart products is presented in Section “Literature Review.” In Section “Hypothesis Development,” the hypotheses and research framework are developed, following which the methodology is proposed in detail in Section “Methodology.” The results are summarized in Section “Results.” Finally, conclusion, implications, limitations, and future research are discussed in Section “Discussion.”

## Literature review

### Individual positive personality

For an individual, positive personality is first described as a healthy personality as the absence of disorder (Millon, 1996). Moreover, it comprises elements beyond mere non-normality or abnormality (Millon, 2003). It is endowed with a value system (Leising et al., 2009). Individual differences in it could be coded in people’s implicit expectations or ideas of how a highly valued individual is like or behaves (de la Iglesia and Castro Solano, 2018). From an empirical perspective, it can be defined as a collection of traits that positively associate with individuals’ psychological outcomes, such as increases in happiness and decreases in depressive symptoms, and increments in coping with work stress, work productivity, and wellbeing (Wagner and Ruch, 2015; Lavy and Littman-Ovadia, 2017; de la Iglesia and Castro Solano, 2018).

For its components, Leising et al. (2009) suggested a 10-cluster solution, that is, independence, tolerance, self-confidence, self-control, friendliness, fairness, trustworthiness, openness, convention, and finally looking for the good in people. In the high five model proposed by Cosentino and Castro Solano (2017), positive personality covers erudition, peace, cheerfulness, honesty, and tenacity. Similarly, de la Iglesia and Castro Solano (2018) also proposed a positive personality model with five dimensions, namely, wellbeing, positive bonds, humanity, moderation, and lucidity.

Nevertheless, the most popular personality theory is Big Five. It separates an individuals’ personality into five dimensions, namely, extroversion, agreeableness, conscientiousness, neuroticism, and openness to experience (Plaisant et al., 2010). Each dimension has two functional sides: the positive one and the reverse. In prior empirical studies, extroversion, agreeableness, conscientiousness, and openness were often considered positive personalities, showing positive effects on expected outcomes, such as creativity (Kaspi-Baruch, 2019; Hong et al., 2020), academic achievement (Wang et al., 2011), and work performance (Zell and Lesick, 2021). However, neuroticism is an exception. Nie et al. (2008) found that neuroticism is negatively associated with most social adaptive behaviors, while other dimensions play significantly positive roles. Sharpe et al. (2011) verified the negative effect of neuroticism on optimism. Lamers et al. (2012) stated that neuroticism is related to psychopathology. Steel et al. (2008) showed the strong relationship between neuroticism and depression, as well as anxiety. According to the aforementioned evidence, the study focuses on the exploration of the relationship between consumers’ four positive personalities (excluding neuroticism) and the purchase behavior of smart products.

### Purchase behavior of smart products

The technology acceptance model and planned behavior theory are often used to explain the mechanism of smart product purchase behavior. In the technology acceptance model, consumers are divided into early adopters and successors. Early adopters’ purchase decision-making determined by aesthetic and utilitarian product-related factors (e.g., design appeal and perceived usefulness), socio-cultural factors (e.g., subjective norms and face considerations), and brand-related factors (brand popularity, perceived brand quality, and brand loyalty) is of crucial importance to the market development of new smart products (Filieri et al., 2017). The purchase behaviors of successors are induced by the recommendation of early adopters through communication in social networks. According to the planned behavior theory, perceived behavioral control, attitude, moral norms, and environmental concern would jointly affect the intention to purchase smart products (Aslam et al., 2021). In the context, technological and product-related factors, such as perceived usefulness and perceived ease of use constitute the antecedents of behavioral attitude, and environmental, social, and psychological factors enable moral norms and trust belief on smart products (Li and Kim, 2016).

Many factors, along with value chain, influence consumers’ purchase intention and behavior of smart products (Shin et al., 2018). First, manufacturers’ technological level would affect the smartness (autonomy, adaptability, and reactivity), usability (compatibility, observability, and complexity), and packaging (anti-counterfeit, recycle, and traceability) of their smart products, which are associated with the psychological factors of purchase decision, such as consumers’ perceived value, risks, and experiences (Kumar et al., 2008; Rijsdijk and Hultink, 2009). Second, the features such as enjoyment, perceived usefulness, and perceived ease of use have a significant impact on consumers’ purchase behavior of smart products (Shin and Ju, 2015; Ahn, 2016). Third, product brand personality and some marketing tools (e.g., advertisement) could play roles in the improvement of consumers’ perception of product value (Lee et al., 2017; Woo and Kim, 2022). Moreover, individual characteristics such as innovation, fashion, leadership, self-efficacy, and concern for health and time determine the purchase intention and consuming behavior (Ko et al., 2009; Shin and Ju, 2015). Finally, consumers’ information search behavior and their knowledge about the product, context, and environment are also important factors of purchase decision (Wang and Chen, 2018; Wang et al., 2022), and so the improvement of consumers’ sociality and training may benefit to the marketing of smart products (Ahn, 2016).

## Hypothesis development

### The generation of purchase intention

Consumers’ purchase intention is generated from the psychological cognition and evaluations for smart products (Woo and Kim, 2022). Product knowledge and information provide foundations for consumers’ accurate cognition and positive evaluation (Brucks, 1985). Consumer knowledge supports the willingness and attitudes of consumers toward specific products (Musova et al., 2021). People rely on their existing knowledge and contextual information to make purchase decisions (Philippe and Ngobo, 1999). There are two kinds of knowledge involving products, that is, objective and subjective knowledge (Huang and Fan, 2002). The former refers to various technological and functional parameters of products. The latter refers to consumers’ cognition and perception of products on the basis of their own values. Because of the asymmetric information between manufacturers and consumers, most consumers lack sufficient knowledge about smart products that possibly involve high technology. A discrepancy always exists between the two kinds of knowledge (Hoque and Alam, 2020), that is, what consumers think they know about a product and what they actually know about it are often different (Schacter, 1983). Hence, in the decision of smart product purchase, consumers’ subjective knowledge often plays the dominant role. Park and Lessig (1981) hold that subjective knowledge based on perception and confidence can better reflect consumers’ decision patterns and predict their purchase behaviors.

In addition to product knowledge, cognitive ability also plays an important role in the generation of consumers’ purchase intention. Cognition enables individuals to receive, select, and process information, as well as transfer the information into valuable knowledge for decision-making (Toomey and Heo, 2019). According to prior studies, cognitive ability assists consumers to generate purchase intention through three paths: First, it raises consumers’ cognitive trust toward certain smart products (Dadzie et al., 2018); second, it improves consumers’ emotion, feeling, and self-confidence, which stimulate their desires for enjoyable and advanced smart products (Cho and Koo, 2014). In the cognitive decision-making algorithms proposed by Kliestik et al. (2022a), consumer sentiments were identified as important factors that influence consumers’ choices and shopping behaviors; third, it increases consumers’ effective responses to social contact, which may trigger impulsive purchase (Sohn, 2013). A proportion of purchase behaviors is actually induced by consumer engagement and experience (Kliestik et al., 2022b). Barbu et al. (2021) have confirmed that cognitive experience, affective experience, and social experience are all positively associated with customers’ loyalty intention.

In this study, we summarize the product knowledge and cognitive ability as consumer knowledge. Several studies have tested the relationship between consumer knowledge and purchase intention. For example, Hwang (2015) verified the positive impacts of consumers’ subjective knowledge, product knowledge, and the cognition of the brand image on purchase intention. Xiao et al. (2021) proposed that consumer knowledge and environmental perception have a positive effect on attitude and, in turn, the intention to purchase green products. Moreover, Boshoff et al. (2011) stated that the cognition of the brand significantly mediates the impact of risk perceptions on the intention to online purchase. Andronie et al. (2021) constructed a neuromanagement decision-making model in which the technological adoption of mobile commerce apps is explained and solved by a cognitive algorithmic process.

We therefore propose the following hypothesis:

H1: Consumer knowledge is positively associated with the intention to purchase smart products.

### The relationship between positive personality and consumer knowledge

Overall, four positive personalities accelerate the creation, learning, sharing, and use of consumer knowledge. First, extroverted personality enhances consumers’ social skills, which are beneficial to the information exchange and knowledge learning in real and virtual social environments (Chang et al., 2013). Second, agreeable personality makes persons unambitious, altruistic, and trustworthy. They can improve knowledge sharing between consumers and others (Matzler et al., 2011). Third, open personality reflects as the traits of creativity, imagination, and aesthetic, which assist consumers to create and innovate new knowledge (Nakano et al., 2013). Fourth, conscientious personality enables consumers to positively search, sufficiently share, and use knowledge to make right purchase decisions (Obrenovic et al., 2022). In addition, individual values and beliefs related to positive personality play an important role in the processes of selecting, integrating, and creating subjective knowledge. As proposed by Nica et al. (2022), consumers’ values generate and lead their cognition and attitudes in the process of shopping decision-making. In addition, previous studies have stated the relationship between positive personality and cognitive ability (Curtis et al., 2015; Rammstedt et al., 2016; Carretta and Ree, 2018). Hence, a hypothesis is presented as follows:

H2: Positive personality is positively associated with consumer knowledge.

### The mediating role of consumer knowledge

Researchers in cognitive school always highlight the important role of knowledge and cognitive ability in the mechanism of intrinsic motivation (Colquitt et al., 2000). They argue that individuals’ intentions and behaviors are determined by intrinsic factors, such as thought, mentality, interest, value, and need (Arshad et al., 2021). The factors arouse individuals’ intentions and stimulate their behaviors under certain environmental conditions. According to the theory of personality dynamic, those factors are involved in individuals’ personalities (Boag, 2018). Personality is the root of individuals’ complex and flexible thoughts, emotions, and behaviors. In the context of our study, consumers with positive personalities would lead themselves to embrace new smart things, search for more information about smart products, and, in turn, arouse the desire to purchase and use smart products. It constitutes a whole process of positive self-motivation. Moreover, consumer knowledge could improve the performance expectancy of smart products, including perceived usefulness, relative advantage, and outcome expectations, which arouses and strengthens consumers’ hedonic motivation hidden in positive personality, thus causing the intention and behavior of purchase in turn (Vinerean et al., 2022). Leu (2008) has discussed the positive correlation between consumers’ personality characteristics and senses toward cell phone brands and proposed that consumers’ behavioral decision is determined by their personalities and psychological levels. McEachern and Warnaby (2008) found that value-based labels could significantly promote consumers’ purchase behaviors when the labels effectively transfer knowledge to customers and even bring them openness to experience (i.e., validated personality traits related to intellectual capability). We thereby present a new hypothesis as follows:

H3: Consumer knowledge positively mediates the impact of positive personality on the intention to purchase smart products.

### The relationship between purchase intention and purchase behavior

As the theory of planned behavior proposed, an individual’s actual behavior can be reasonably inferred from behavioral intention, which is determined by attitude, subjective norm, and perceived behavioral control based on beliefs (Bleakley and Hennessy, 2012). The intention to purchase a specific product has been found to be a good predictor of actual purchase behavior (Ramayah et al., 2010). For example, Kamalanon et al. (2022) showed that green purchase intention positively and significantly affects green purchase behavior. Mazhar et al. (2022) also found that green purchase intention stimulates green food behavior. However, the information retrieval by authors indicates that few scholars have paid attention to the relationship between psychological intention and actual behavior in purchasing smart products. Hence, we think that the argumentation of the following proposed hypothesis is necessary.

H4: Consumers’ purchase intention is positively associated with the behavior in purchasing smart products.

### The mediating role of purchase intention

In the ABC model of attitudes, the relationships between affect, behavior, and cognition are discussed (Liu et al., 2021). Comparing to the theories toward behavioral formation, the model proposes that the attitude to an object is the combination of cognition (knowledge and ability), affect, and behavior so as to better predict the adoption of new things, such as various smart products (Solomon, 1996). The model has been used in various research areas, especially consumer behavior (Callender, 2015). According to the model, consumers at the standard learning hierarchy form affective preferences for products based on existing cognition and knowledge and then generate behavior toward purchase decision (Caro and Garcia, 2007). As proposed by Zibret et al. (2018), consumers’ subjective knowledge results in a significant effect on purchase intention, and the intention and consumers’ perceived self-efficacy can significantly predict the purchasing frequency. We thereby developed the following hypothesis:

H5: Purchase intention positively mediates the impact of consumer knowledge on purchase behavior.

### The relationship between positive personality and perceived income

Perception is an important variable in customer psychology research. Consumers’ perceptions would affect their expectations of products and, in turn, determine their decisions, behaviors, and even habits (Hopkins, 2022). A consumer’s perceived income is the subjective perception of the individual’s absolute and relative income level. People often compare their actual income with some reference standards, such as the income of peers or their previous income level (Ucal and Gunay, 2019). Easterlin (1974) suggested that differences in relative, rather than absolute, income levels may better explain people’s consuming behaviors.

Individuals’ positive personality affects perceived income from two aspects: First, prior studies have proved that positive personalities including extroversion, agreeableness, conscientiousness, and openness to experience are all positively associated with the individuals’ inputs and outcomes, such as work engagement (Lv et al., 2018), work competence (Hoffman and Woehr, 2006), career satisfaction and success (McCann, 2018; Semeijn et al., 2020), and job performance (Mammadov, 2022). Denissen et al. (2018) proposed that to some extent, economic success depends on “having a successful personality.” Similarly, as the report of Judge and Zapata (2015) suggests, positive personality is a good predictor for a person’s earnings. The high level of a person’s actual income lays the foundation of the individual’s experience in income and happiness. On the contrary, Yoon et al. (2015) proposed that the type D personality characterized by negative affect and social inhibition is an important factor for a person’s low income and even causes more serious consequences, such as suicide.

Second, positive personalities are related to the perception of income inequality (de Vries et al., 2011). People with positive personalities are often less greedy about their income levels. They are more willing to be satisfied with their *status quo* and adapt expected expenditure when their actual incomes reduce. In addition, people with positive personality traits tend to be more active and self-confident in life, work, and social life, which reduce their focuses on income comparisons and raise their life satisfactions (Budria and Ferrer-I-Carbonell, 2019). Hence, we propose the following hypothesis:

H6: Consumers’ positive personality is positively associated with perceived income.

### The relationship between perceived income and purchase behavior

Consumers make decisions to purchase products in line with their income levels, aesthetics, and statuses or positions, among which the level of actual income is often regarded as a restrictive factor of consumption. The same consumption decisions are possibly deemed less permissible for a lower income individual than for an individual with higher or uncertain income (Hagerty and Barasz, 2020). Persons with low perceived income would segregate themselves from the consumption of luxurious and unessential products. A portion of smart products focusing on providing intelligent service and smart experience, rather than meeting necessary requirements in life, is therefore listed in segregated inventory (Hagerty and Barasz, 2020). In addition, the enhancement of perceived income would improve individuals’ self-cognition of identity and social position. The cognition further affects individuals’ behavior to decide what to buy. Sun et al. (2021) found that consumers’ behavior of purchasing iPhone is dominated by social comparison and the need for uniqueness, which are often pursued by the consumer group with a relatively high perceived income. However, Antiniene et al. (2021) have drawn a conclusion that regarding consumers’ perceived relative income, Lithuanians with a low income are more prone to aspire to material possessions, causing overconsumption behaviors. We propose the following hypothesis:

H7: Consumers’ perceived income is positively associated with the behavior to purchase smart products.

### The mediating role of perceived income

Through comparisons, people with different personality traits may have different perceptions of their actual incomes (absolute income and relative income). The surplus perceived income compared to expectation stimulates the improvement of the consumption level, while the deficit of perceived income strengthens perceived risk, causing to cut unnecessary expenditure and increase savings (Shin and Kim, 2018). As proposed by Oshio and Urakawa (2014), individuals with different kinds of personality traits perceive different degrees of income inequality, which influence their consumption levels, living status, and subjective wellbeing. Considering smart products’ characteristics, generally expensive, alternative, and non-essential, we argue that the purchase budget and the decision on what type of smart products in the same category to buy are highly sensitive to perceived income. In the model constructed by Parag and Butbul (2018), consumers’ perceived income and openness to experience jointly explained their interest in smart homes. Consequently, we develop the following hypothesis:

H8: Consumers’ perceived income positively mediates the impact of positive personality on purchase behavior.

### Research framework

**FIGURE 1:**
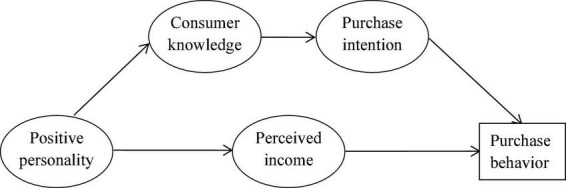
Research framework of our study.

Personality traits have long been shown to contribute to consumer behaviors (Liu et al., 2017). As a matter of fact, behaviorists hold the view that individuals’ personalities matter all of their behaviors, including purchase decision-making. However, the trigger mechanism of consumer behavior to purchase smart products has not been deeply explored. In the study of Arshad et al. (2021), a research framework for individual intention was developed based on the motivational theory. It proposed two kinds of motivational factors, namely, intrinsic factors (e.g., cognition and interest) and extrinsic factors (e.g., perceived income). Guided by the behaviorism theory, we use the framework for reference to link the aforementioned hypotheses and then construct the research framework of our study, as shown in Figure 1.

## Methodology

### Measures

The variables include positive personality (extroversion, agreeableness, conscientiousness, and openness), consumer knowledge (product knowledge and cognitive ability), purchase intention, perceived income, and purchase behavior. We make a survey to collect the data of these variables. A questionnaire is developed for the survey. In the questionnaire, variables (with the exception of demographic variables and purchase behavior) are measured by a five-point Likert scale. Respondents are required to rate the items from “1 strongly disagree” to “5 strongly agree.”

The items of four positive personalities are selected from the Big Five Inventory developed by John et al. (1991), with a total of 15 items, including “I enjoy talking to people” and “I try to be courteous and polite to everyone I meet.”

Totally, 12 items are selected for measuring consumer knowledge, and six items, such as “I know a lot about smart products” and “I always pay attention to new smart products,” are adapted from Cai et al. (2016) to measure objective product knowledge. Another six items, including “I can always analyze the strengths and weaknesses of things quickly” and “I can collect information quickly to assist my decision making,” are learned from Mannucci and Yong (2018) to measure cognitive ability.

Consumers’ purchase intention refers to Xing (2020) and Zhou et al. (2021), with six items including “I prefer to consume smart products over traditional products” and “I always keep an eye on certain smart products that I am interested in.”

The measure of perceived income refers to Meng et al. (2015). It includes three items, such as “Compared with my peers, I am very satisfied with my income” and “Compared with the average personal income in my region, my income is very high.”

Unlike the previous mentioned constructs, purchase behavior is measured by three quantitative items, namely, in the past year, (a) consumer’s expenditure on smart products, (b) the ratio of expenditure on smart products to total income, and (c) the type number of smart products the consumer has purchased. After data collection, we process the data of each item into intervals [1,5] so as to better match the data of qualitative variables. Finally, we assign the mean value of three items to purchase behavior.

### Data

Questionnaires were issued through Sojump^1^, a professional online platform for survey service in China. Many Chinese scholars have used it during their academic research processes (e.g., Li and Yu, 2018). The data were collected from October 6 to 21, 2021. After manual screening to eliminate invalid questionnaires, we retrieved 326 valid questionnaires, with a valid recovery rate of 84.7%.

After statistics, the respondents account for 46.93% male and 53.07% female participants. The respondents are concentrated in the age of 18∼40 years, of which 28.83% are 18 to 25 years old, 30.37% are 26 to 30 years old, 27.61% 31 to 40 years old, 7.67% 41 to 50 years old, and finally 5.52% are 51 or above. The education level of the respondents is generally high, with 40.18% of them having a master degree or above, 48.47% having a bachelor degree, 3.37% graduated from high schools, and 7.98% graduated from junior schools or below. Respondents are mainly from 12 different occupations, such as school students (29.14%), engineers (18.1%), teachers (15.03%), and civil servants (5.83%). The distribution of respondents’ monthly income is as follows: 28.22% earn below 2000 yuan, 21.47% between 2000 and 5000 yuan, 20.25% between 5001 and 8000 yuan, 18.4% between 8001 and 12000 yuan, and only 11.66% earn above 12001 yuan. Through identifying their IP address, we identified that the respondents live or work in 26 (total 31) different Chinese provinces.

### Common method bias

In order to control common method bias, we rearranged the items in the questionnaire and required the respondents to fill it anonymously. After data collection, we used the Harman one-way method to extract the principal components with an eigenvalue greater than 1. The cumulative variance explained by the first component is 29.057%, which is less than the threshold value of 40%. Hence, the potential problem of common method bias has been effectively controlled.

### Reliability and validity

In the study, the reliability and validity were tested by IBM SPSS Statistics 26 and Smart PLS 3.0. Cronbach’s alpha of total constructs as a whole is 0.924, which is greater than 0.7, indicating a high internal consistency reliability. The KMO value calculated by exploratory factor analysis is 0.907, which is greater than 0.7, indicating the existence of common factors among variables. The significance of Bartlett’s sphericity test is 0.000, which is less than 0.05, indicating that the data are suitable for factor analysis. The results of confirmatory factor analysis (CFA) and exploratory and factor analysis (EFA) are shown in Table 1.

**TABLE 1:** EFA and CFA results.

Construct	Item no.	Factor loading		VIF	*p*-value
		EFA	CFA		
Extroversion	1	0.761	0.759	1.468	0.000
	2	0.800	0.801	1.971	0.000
	3	0.879	0.879	2.421	0.000
	4	0.769	0.770	1.633	0.000
Openness	1	0.761	0.761	1.285	0.000
	2	0.724	0.725	1.291	0.000
	3	0.847	0.847	1.451	0.000
Agreeableness	1	0.775	0.774	1.369	0.000
	2	0.796	0.796	1.335	0.000
	3	0.804	0.805	1.418	0.000
Conscientiousness	1	0.702	0.703	1.441	0.000
	2	0.756	0.756	1.548	0.000
	3	0.740	0.738	1.486	0.000
	4	0.698	0.698	1.378	0.000
	5	0.739	0.739	1.512	0.000
Purchase intention	1	0.776	0.776	1.834	0.000
	2	0.755	0.755	1.719	0.000
	3	0.799	0.799	2.060	0.000
	4	0.856	0.856	2.344	0.000
	5	0.674	0.674	1.475	0.000
	6	0.612	0.612	1.357	0.000
Perceived income	1	0.889	0.889	2.160	0.000
	2	0.877	0.876	2.198	0.000
	3	0.724	0.725	1.295	0.000
Product knowledge	1	0.750	0.750	1.770	0.000
	2	0.805	0.805	2.429	0.000
	3	0.836	0.836	2.688	0.000
	4	0.819	0.819	2.136	0.000
	5	0.761	0.761	1.721	0.000
	6	0.714	0.714	1.565	0.000
Cognitive ability	1	0.650	0.650	1.447	0.000
	2	0.757	0.757	1.716	0.000
	3	0.836	0.836	2.207	0.000
	4	0.780	0.780	1.849	0.000
	5	0.708	0.708	1.647	0.000
	6	0.838	0.838	2.300	0.000
Consumer knowledge	Product knowledge	0.907^a^		1.478	0.000
	Cognitive ability	0.862^a^		1.478	0.000

^a^Factor weight of formative constructs.

The structural equation model constructed in our study contains both reflective and formative constructs. Factor loading is used to judge the discriminate validity of reflective constructs, and factor weight is suitable for formative constructs. As shown in Table 1, the results of CFA and EFA are consistent with each other. The factor loadings of most constructs in the study are greater than 0.707 (an exception exists in two items of purchase intention; their factor loadings are 0.674 and 0.612, respectively), meeting the criteria. Chin (1998) argued that the factor weights of formative constructs need to be greater than 0.2 and significant. Obviously, the factor weights of formative constructs in our study are all greater than the recommended value. In addition, the *p*-values of all items calculated through the bootstrap process are all less than 0.001, meeting the requirement of the significance level. Moreover, the variance inflation factor (VIF) is used to check multicollinearity. Hair et al. (2017) recommended that the VIF should be smaller than 5. In our study, all VIF values meet requirements.

The construct reliability and validity are shown in Table 2. Cronbach’s alpha of each construct is greater than or close to 0.7, indicating the good reliability of the scale. From the table, we can find that the values of composite reliability (CR) are all greater than the recommended value 0.8, and the values of average variance extracted (AVE) are all greater than 0.5. Both of them prove that the constructs in our model have good convergent validity.

**TABLE 2:** Construct reliability and validity.

Construct	Cronbach’s alpha	CR	AVE
Extroversion	0.816	0.879	0.646
Openness	0.676	0.822	0.607
Agreeableness	0.703	0.834	0.627
Conscientiousness	0.777	0.849	0.529
Purchase intention	0.841	0.884	0.562
Perceived income	0.776	0.871	0.694
Product knowledge	0.872	0.904	0.612
Cognitive ability	0.856	0.893	0.584

We used two approaches to measure discriminant validity. First, we calculated the square root of AVE values and the correlations between every two constructs. The results are shown in Table 3. It demonstrates that each square root of AVE is greater than the correlations between the corresponding construct and other constructs. Second, we calculated the heterotrait-to-monotrait ratio (HTMT) of each construct. As shown in Table 4, the HTMT of most constructs is less than the strict recommended value of 0.85, and the exception is the HTMT between agreeableness and conscientiousness is 0.859, which is less than the loose recommended value of 0.90. Hence, our constructs have good discriminant validity.

**TABLE 3:** Square root of AVE and correlations.

Construct	1	2	3	4	5	6	7	8
1. Extroversion	**0.804**							
2. Openness	0.525	**0.792**						
3. Agreeableness	0.465	0.636	**0.727**					
4. Conscientiousness	0.441	0.455	0.549	**0.779**				
5. Purchase intention	0.407	0.277	0.292	0.453	**0.750**			
6. Perceived income	0.270	0.230	0.130	0.087	0.120	**0.830**		
7. Product knowledge	0.354	0.223	0.196	0.378	0.700	0.270	**0.780**	
8. Cognitive ability	0.511	0.359	0.378	0.407	0.530	0.300	0.570	**0.764**

The bold diagonal values are the square roots of AVE.

**TABLE 4:** Heterotrait-to-monotrait ratio.

Construct	1	2	3	4	5	6	7	8
1. Extroversion								
2. Openness	0.577							
3. Agreeableness	0.674	0.652						
4. Conscientiousness	0.576	0.749	0.859					
5. Purchase intention	0.497	0.605	0.366	0.372				
6. Perceived income	0.346	0.126	0.314	0.177	0.148			
7. Product knowledge	0.416	0.485	0.286	0.241	0.814	0.322		
8. Cognitive ability	0.618	0.528	0.462	0.466	0.623	0.369	0.650	
9. Purchase behavior	0.085	0.186	0.075	0.046	0.355	0.229	0.454	0.210

Finally, for the correlations in Table 3, we still need to address three things: First, all intercorrelations, including the greatest correlation 0.700 between product knowledge and purchase intention, meet the requirement of the recommended value (less than 0.9), indicating the validity of our measurements; second, the high correlations between several pairs of constructs just support our hypotheses preliminarily; and third, the possible multicollinearity problems accompanied by high correlations have been effectively controlled, as shown in Table 1.

### Modeling methods

In our study, the partial least square-based structural equation model (PLS-SEM) was used to test the hypotheses. The tool for data analysis is Smart PLS 3.0. Compared with covariance based the SEM, the PLS-SEM can handle complex models with both formative and reflective constructs, suitable for our proposed model.

Researchers have proposed several methods to measure the higher order PLS-SEM, such as indicators reuse method and two-stage method (Hair et al., 2017). The first one is more applicable to reflective–reflective higher order structures. Hence, it was used to measure the structure of consumer positive personality. The second one is fit for reflective–formative higher order structures (Wetzels et al., 2009). Existing studies have proposed two two-stage approaches: the embedded one (Ringle et al., 2018) and the disjoint one (Becker et al., 2012). The two have slight differences, but they often produce similar results (Cheah et al., 2018). We chose the embedded two-stage approach to measure consumer knowledge. In the first stage, in line with indicators reuse approach, we modeled the entire higher order structure to estimate the non-significant coefficients between lower order components and higher order components. The scores of higher order components were saved as new variables and added into our data set. In the second stage, the scores obtained in the first stage were used as the data of latent variables in higher order model.

## Results

**FIGURE 2:**
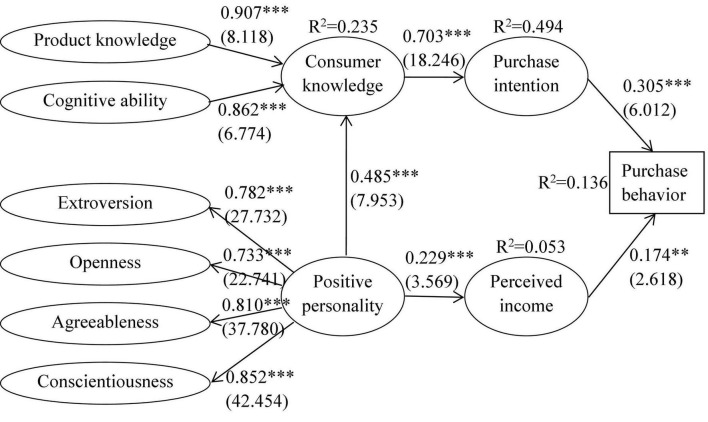
Estimation results of PLS-SEM. ^***^p < 0.001, ^**^p < 0.01. The data inside the parentheses are *t*-statistics.

Using Smart PLS 3.0, we successfully estimated all coefficients of the PLS-SEM. The results are shown in Figure 2, in which the significance values were calculated through the bootstrap process (bootstrap = 5000). As shown in the figure, most *R*^2^ values of the models are relatively strong, indicating good goodness of fit. In addition, the *Q*^2^ values of all models calculated by using the blindfolding method are greater than 0, indicating good predictive relevance. In detail, the effect of positive personality on consumer knowledge is 0.485 (*p* < 0.001), and the effect of consumer knowledge on purchase intention is 0.703 (*p* < 0.001), and hypotheses H1 and H2 are therefore supported. Moreover, the effect coefficient between purchase intention and purchase behavior is 0.305 (*p* < 0.001), indicating that hypothesis H4 passes the test. In addition, the effects of positive personality on perceived income (estimate = 0.229, *p* < 0.001) and of perceived income on purchase behavior (estimate = 0.174, *p* < 0.001) are also significant. Hence, hypotheses H6 and H7 are accepted.

Employing the bootstrap process, we tested the mediating effects in our hypotheses. The results are summarized in Table 5. First, the indirect effect of consumer knowledge on the relationship between positive personality and purchase intention is 0.341 (*p* < 0.001). Hypothesis H3 is therefore supported. Second, the mediating effect of purchase intention on the path from consumer knowledge to purchase behavior is 0.214 (*p* < 0.001), which supports hypothesis H5. Third, the mediating effect of perceived income on the relationship between positive personality and purchase behavior is also significant (estimate = 0.040, *p* < 0.05). Hypothesis H8 is therefore accepted.

**TABLE 5:** Mediating effects.

Indirect path	Indirect effects	Standard deviation	*T*-statistics	*p*-value
Positive personality → Consumer knowledge → Purchase intention	0.341	0.053	6.403	0.000
Consumer knowledge → Purchase intention → Purchase behavior	0.214	0.039	5.489	0.000
Positive personality → Perceived income → Purchase behavior	0.040	0.016	2.562	0.010

All in all, our results show that the direct and indirect effects on both affecting paths are all significant. However, the total effect in the path throughout consumer knowledge and purchase intention (estimate = 0.104, *p* < 0.001) is much stronger than the total effect generated in the path *via* perceived income (estimate = 0.040, *p* < 0.05).

## Discussion

### Conclusion

In our study, two mechanisms have been explored to explain and predict the effect of consumer positive personality on purchase behavior. The first one is the chain mediated by consumer knowledge and purchase intention in sequence. It highlights the dominant important role of consumer positive personality in purchasing behavior and proposes the cooperative governance of consumers’ positive personality and knowledge when retailers manage their customers. The second one is mediated by perceived income, which is recognized as an important socioeconomic factor that may magnify or restrain individual desires. It is a novel theoretical idea that smart product purchasing behavior is jointly predicted by the two, contributing new knowledge to the literature. In addition, compared with the strong indirect effect generated in the first mechanism, the effect of the second mechanism is much weaker. It may attribute to the fact that most of our samples are young people. Their desires for smart products cannot be effectively restrained by their relatively weak perception of current income.

In the context of smart product purchase, two kinds of knowledge are required for consumers to make decisions, that is, subjective product knowledge and general cognitive ability. Although scholars have highlighted the separate roles of product knowledge and cognitive ability in the occurrence of consumers’ purchase behavior (Shih, 2012; Ahn, 2016; Kim, 2021b), we developed a study where we incorporated them into a model, which enriched our understanding of the structure of consumer knowledge. Moreover, in the study of Testa et al. (2019), the effect of consumer product knowledge on actual purchase behavior mediated by intention has been measured in the context of organic food consumption. In that study, the estimated effects between consumer product and purchase intention, and between purchase intention and actual behavior are 0.1797 (*p* < 0.05) and 0.6535 (*p* < 0.001). By contrast, the effects of those in our study are 0.703 (*p* < 0.001) and 0.305 (*p* < 0.001), respectively, indicating that (a) consumer knowledge is more important in the purchasing decision of smart products relative to organic food, and (b) comparing to the decision-making in organic food purchase, the decision-making for purchasing smart products would be more careful; therefore, the link between purchase intention and actual behavior is much weaker.

We defined and measured positive personality based on the Big Five theory. The measurement is mature, with high reliability and validity, and could be popularized and applied to similar research scenarios. In the study, extroversion, agreeableness, conscientiousness, and openness are essential components of individual positive personality, but neuroticism is an exception. The knowledge is similar to the viewpoints of Sharpe et al. (2011) and Kim (2021a). In addition, the ideas and methods developed in our study could be expanded into other fields of marketing, such as the exploration of the trigger mechanism of behaviors for purchasing green food, luxury products, or artworks.

### Theoretical implications

The theoretical contributions of this study are mainly in three aspects. First, the construction of the research framework for explaining the occurrence of consumer behavior involving smart product purchase from twofold perspectives enriches the theory of consumer psychology and behavior. It arouses researchers to pay more attention to the role of positive personality in the generation of purchase intention and behavior. It also reminds to think twice about the dynamic and complex mechanism of purchase behavior from psychological perspective. Second, the measurement of consumer positive personality from the aspects of extroversion, agreeableness, conscientiousness, and openness expands the applied range of the Big Five theory. In future, scholars can take this approach to measure individual positive personalities. Third, the measurement of consumer knowledge from the aspects of product knowledge and cognitive ability is also a theoretical innovation. This kind of definition for consumer knowledge improves our understanding of the components and structure of consumers’ knowledge for decision-making related to purchase and consumption.

### Practical implications

The practical implications of the study can be summarized in four aspects. First, from consumers’ perspective, individuals with positive personality could sufficiently exploit the advantage to the full so as to help them make right purchase decisions. They are also warned to reasonably control their consumption over their perceived incomes. Second, from retailers’ perspective, retail stores are recommended to construct a system for customer relation management, a part content of which is to intelligently distinguish customers’ positive personality. Shop assistants are recommended to get a professional training related to the communication with customers with different types of personality. Third, from the perspective of the manufacturers of smart products, they are required to consider more about consumers’ personality, product knowledge, and income level when they devote effort into the development of new smart products. Moreover, the right construct of brand personality for their smart products, in accord with the personality of targeted customers, is a good idea. Finally, from data developers’ perspective, the data collection and analysis of individuals’ personality have a huge market prospect. All manufacturers, agents, and retailers would be the potential customers. The data have wide applications with great value in the fields, such as new product development, advertisement, and customer relation management.

### Limitations and future research

Some limitations do exist in our study. First, the cross-sectional data collected by us cannot infer the causal relationships as a matter of fact. We need to collect longitudinal data in future. Second, the samples limited in Chinese consumers restrain the generalizability of our results to a certain extent. Future research should pay more attention to cross-cultural analyses. Third, the sample size meets the requirement of model estimation, but we also need to expand our sample size to gain more robust results. Fourth, more control variables need to be introduced into the model, in order to better control the possible influences of individual characteristics, contextual factors, and economic factors on the consumer behavior of purchasing smart products. Finally, future research could consider substituting the components of positive personality, since the connotation of positive personality is abundant and has been no consensus yet.

Regardless of the limitations described before, our study highlights some possible future research directions. For instance, in order to enrich the theory, we can introduce other potential mechanisms of the stimulation of consumers’ behaviors of smart product purchase. Moreover, scholars could employ and develop the framework of our study to predict the purchase behavior in broader fields.

## Data availability statement

The raw data supporting the conclusions of this article will be made available by the authors, without undue reservation.

## Ethics statement

Ethical review and approval was not required for the study on human participants in accordance with the local legislation and institutional requirements. The patients/participants provided their written informed consent to participate in this study.

## Author contributions

DY designed and performed the research, checked the results, and improved the manuscript. DL processed the data and wrote the draft. Both authors reviewed, edited, and approved the final manuscript.

## Footnotes

1
http://www.sojump.com
